# Author Correction: A cross-cohort replicable and heritable latent dimension linking behaviour to multi-featured brain structure

**DOI:** 10.1038/s42003-023-04697-2

**Published:** 2023-03-17

**Authors:** Eliana Nicolaisen-Sobesky, Agoston Mihalik, Shahrzad Kharabian-Masouleh, Fabio S. Ferreira, Felix Hoffstaedter, Holger Schwender, Somayeh Maleki Balajoo, Sofie L. Valk, Simon B. Eickhoff, B. T. Thomas Yeo, Janaina Mourao-Miranda, Sarah Genon

**Affiliations:** 1 Institute of Neuroscience and Medicine (INM-7: Brain and Behaviour), Research Centre Jülich, Jülich, Germany; 2 Centre for Medical Image Computing, Department of Computer Science, University College London, London, UK; 3 Max Planck University College London Centre for Computational Psychiatry and Ageing Research, University College London, London, UK; 4 Department of Psychiatry, University of Cambridge, Cambridge, UK; 5 Institute of Systems Neuroscience, Heinrich Heine University Düsseldorf, Düsseldorf, Germany; 6 Mathematical Institute, Heinrich Heine University Düsseldorf, Düsseldorf, Germany; 7 Otto Hahn Research Group “Cognitive Neurogenetics”, Max Planck Institute for Human Cognitive and Brain Sciences, Leipzig, Germany; 8 Department of Electrical and Computer Engineering, Centre for Translational MR Research, Centre for Sleep & Cognition, N.1 Institute for Health, Institute for Digital Medicine, National University of Singapore, Singapore, Singapore

**Keywords:** Cognitive neuroscience, Human behaviour

Correction to: *Communications Biology* 10.1038/s42003-022-04244-5, published online 26 November 2022.

In the original version of this Article, the labels provided for brain loadings in Supplementary Data 2 and Supplementary Data 4 corresponded to an outdated version of the Schaefer 200 granularity atlas. These files have now been updated with the current labels for this atlas. The html version of this article has been corrected.

## Supplementary information

Updated Supplementary Data 2

Updated Supplementary Data 4

