# Supplementary material for: A cross-cohort replicable and heritable latent dimension linking behaviour to multi-featured brain structure
Source: Commun Biol. 2022 Nov 26;5:1297. doi: 10.1038/s42003-022-04244-5 (PMC9701210; doi:10.1038/s42003-022-04244-5)
Supplement: Supplementary file 2 — Supplementary Materials [file 42003_2022_4244_MOESM2_ESM.pdf]

A cross-cohort replicable and heritable latent dimension linking behaviour to multi-featured brain structure

Supporting information

Supplementary results

Modular analysis linking behaviour with CT

In the HCP-YA cohort, the modular analysis linking behaviour with CT features showed one significant latent dimension (r_{range}=0.13-0.37; p=0.001-0.12) (Supplementary figure 16). The behavioural loadings of this modular latent dimension were correlated with the behavioural loadings of the global latent dimension of both, the HCP-YA (r=0.99, p<0.001) and the HCP-A cohorts (r=0.66, p<0.001). The CT loadings of this modular latent dimension were significantly correlated with the CT loadings of the global latent dimensions in both, the HCP-YA (r=0.98; p<0.001) and the HCP-A cohorts (r=0.83; p<0.001).

On the HCP-A cohort, the modular analysis linking behaviour with CT found one significant latent dimension (r_{range}=0.29-0.39; p=0.001-0.001) (Supplementary figure 17). The behavioural loadings of this modular latent dimension were correlated with the behavioural loadings of the global latent dimension of both, the HCP-A cohorts (r=0.98, p<0.001) and the HCP-YA cohorts (r=0.61, p=0.005). The CT loadings of this modular latent dimension were significantly correlated with the CT loadings of the global latent dimensions in both, the HCP-A (r=0.98; p<0.001) and the HCP-YA cohorts (r=0.79; p<0.001).

Modular analysis linking behaviour with SA

On the HCP-YA cohort, the modular analysis linking behaviour with SA found one significant latent dimension (r_{range}=0.10-0.30; p=0.001-0.12) (Supplementary figure 18). The behavioural loadings of this modular latent dimension were correlated with the behavioural loadings of the global latent dimension of both, the HCP-YA (r=0.99, p<0.001) and the HCP-A cohorts (r=0.74, p<0.001). The SA loadings of this modular latent dimension were significantly correlated with the SA loadings of the global latent dimensions in both, the HCP-YA (r=0.96; p<0.001) and the HCP-A cohorts (r=0.52; p<0.001).

In the HCP-A cohort, the modular analysis linking behaviour with SA features showed two significant latent dimensions (first latent dimension: r_{range}=0.27-0.42; p=0.001-0.002; second latent dimension: r_{range}= -0.02-0.22; p=0.006-0.65). The first latent dimension (Supplementary figure 19) was significantly correlated with the global latent dimensions at the behavioural (HCP-A: r=0.97, p<0.001; HCP-YA: r=0.84, p<0.001) and SA loadings (HCP-A: r=0.98, p<0.001; HCP-YA: r=0.56, p<0.001). The second latent dimension was not significantly correlated with the global latent dimensions neither at the behavioural nor at the SA loadings (p>0.5).

Modular analysis linking behaviour with GMV

In the HCP-YA cohort, the modular analysis linking behaviour with GMV features showed one significant latent dimension (r_{range}=0.17-0.34; p=0.001-0.069) (Supplementary figure 20). The behavioural loadings of this modular latent dimension were significantly correlated with the behavioural loadings of the global latent dimensions of both, the HCP-YA (r=0.99, p<0.001) and the HCP-A cohorts (r=0.73, p<0.001).

On the HCP-A cohort, the modular analysis linking behaviour with GMV found one significant latent dimension (r_{range}=0.17-0.43; p=0.001-0.041) (Supplementary figure 21). This latent dimension was correlated with the behavioural loadings of the global latent dimensions on both, the HCP-A (r=0.99, p<0.001) and the HCP-YA cohorts (R=0.63, p=0.005).

Socio-economic status and site effects in the latent dimension

The analyses linking behaviour and SES to brain structure yielded 3 significant latent dimensions in the HCP-YA cohort (first latent dimension: r_{range}=0.27-0.43, p=0.005-0.01; second latent dimension: r_{range}=-0.07-0.17, p=0.035-0.999; third latent dimension: r_{range}=0.078-0.020, p=0.04-0.85) and 1 significant latent dimension in the HCP-A cohort (r_{range}=0.26-0.49, p=0.005-0.04). Of those, only the first latent dimension (Supplementary figure 22-24) was

replicated across cohorts, showing significant cross-cohort correlations in the behavioural (r=0.62 , p<0.001), CT (r=0.78, p<0.001) and SA loadings (r=0.46, p<0.001). The second latent dimension in the HCP-YA was significantly correlated with the first latent dimension on the HCP-A only on the SA loadings (r=-0.26, p=0.01) All the remaining comparisons were not significant (p>0.14).

Supplementary tables

Supplementary table 1: Behavioural variables

Category/Domain	Subdomain	Column Header	Measure name	Label
Alertness	Sleep	PSQI_Comp1	PSQI	Subjective sleep quality1
		PSQI_Comp2	PSQI	Sleep latency
		PSQI_Comp5	PSQI	Sleep disturbance
		PSQI_Comp6	PSQI	Use of sleep meds
		PSQI_Comp7	PSQI	Daytime dysfunction
Cognition	Episodic memory	PicSeq_Unadj	Picture sequence memory	Episodic memory
	Executive function/Cognitive flexibility	CardSort_Unadj	Dimensional change card sort	Executive function/Cognitive flexibility
	Executive function/Inhibition	Flanker_Unadj	Flanker inhibitory control and attention task	Executive function/Inhibition
	Language/Reading decoding	ReadEng_Unadj	Oral reading recognition	Language/Reading decoding
	Language/Vocabulary comprehension	PicVocab_Unadj	Picture vocabulary	Language/Vocabulary comprehension
	Processing speed	ProcSpeed_Unadj	Pattern comparison processing speed	Processing speed
	Self-regulation/Impulsivity	DDisc_AUC_200	Delay discounting	Self-regulation/Impulsivity1
		DDisc_AUC_40K	Delay discounting	Self-regulation/Impulsivity2
	Working memory	ListSort_Unadj	List sorting	Working memory
Emotion	Emotion recognition	ER40_CR	Penn emotion recognition test	Emotion recognition - CR
		ER40_CRT	Penn emotion recognition test	Emotion recognition - RT-CR Rev

	Negative Affect	AngAffect_Unadj	NIH Toolbox Anger-Affect Survey	Anger - Irritability/frustration
		AngHostil_Unadj	NIH Toolbox Anger-Hostility Survey	Hostility/cynicism
		AngAggr_Unadj	NIH Toolbox Anger-Physical Aggression Survey	Physical aggression
		FearAffect_Unadj	NIH Toolbox Fear-Affect Survey	Fear
		FearSomat_Unadj	NIH Toolbox Fear-Somatic Arousal Survey	Somatic symptoms of anxiety
		Sadness_Unadj	NIH Toolbox Sadness Survey	Sadness
	Psychological well-being	LifeSatisf_Unadj	NIH Toolbox General Life Satisfaction Survey	Life satisfaction
		MeanPurp_Unadj	NIH Toolbox Meaning and Purpose Survey	Meaning/Purpose
	Social relationships	Friendship_Unadj	NIH Toolbox Friendship Survey	Friendship
		Loneliness_Unadj	NIH Toolbox Loneliness Survey	Loneliness
		PercHostil_Unadj	NIH Toolbox Perceived Hostility Survey	Hostility
		PercReject_Unadj	NIH Toolbox Perceived Rejection Survey	Rejection
		EmotSupp_Unadj	NIH Toolbox Emotional Support Survey	Emotional support
		InstruSupp_Unadj	NIH Toolbox Instrumental Support Survey	Instrumental support
	Stress and Self Efficacy	PercStress_Unadj	NIH Toolbox Perceived Stress Survey	Stress
		SelfEff_Unadj	NIH Toolbox Self-Efficacy Survey	Self-efficacy

ASR: Achenbach Adult Self-Report / PSQI: Pittsburgh Sleep Quality Index

Supplementary table 2. Latent dimensions in the HCP-YA cohort

Level	Split	Canonical correlation (r coefficient)	p-value uncorrected	p-value corrected
1	1	0.31	0.002	0.01*
	2	0.28	0.003	0.015*
	3	0.41	0.001	0.005*
	4	0.25	0.004	0.02*
	5	0.40	0.001	0.005*

Statistical results for the significant latent dimension are shown for each one of the 5 outer splits. P-values are shown as uncorrected and corrected for multiple comparisons using the Bonferroni method over 5 comparisons (corresponding to the 5 outer splits)[2]. Asterisks indicate splits that yielded significant latent dimensions. r: Pearson's correlation.

Supplementary table 3. Latent dimensions in the HCP-A cohort.

Level	Split	Canonical correlation (r coefficient)	p-value uncorrected	p-value corrected
1	1	0.61	0.001	0.005*
	2	0.36	0.001	0.005*
	3	0.40	0.001	0.005*
	4	0.29	0.002	0.010*
	5	0.51	0.001	0.005*
2	1	0.33	0.001	0.005*
	2	0.13	0.078	>0.39
	3	0.19	0.020	0.1
	4	0.23	0.010	0.050*
	5	0.04	0.312	>0.999

Statistical results for the two significant latent dimensions are shown for each one of the 5 outer splits. P-values are shown as uncorrected and corrected for multiple comparisons using the Bonferroni method over 5 comparisons (corresponding to the 5 outer splits)[2]. Asterisks indicate splits that yielded significant latent dimensions. r: Pearson's correlation.

Supplementary table 4. Alases used for different levels of anatomical resolution.

Overall granularity	Granularity of cortex (Schaefer atlas)	Granularity of subcortex (Tian atlas)	Granularity of cerebellum (Buckner/Yeo atlas)
323	100	16 (I)	7
1239	200	32 (II)	7
1267	400	50 (III)	17
1871	600	54 (IV)	17

Atlases used to test the effect of different granularity levels. For the cortex, we used 4 levels of granularity of the Schaefer atlas[3], for the subcortex we used 4 levels of granularity of the Tian atlas[4], and for the cerebellum we used 2 levels of granularity from the Buckner/Yeo atlas[5].

Supplementary table 5. Results of latent dimensions with different anatomical resolutions.

Cohort	Granularity	Levels	HCP-A granularity 1239			HCP-YA granularity 1239		
			r	p-value uncorrected	p-value corrected	r	p-value uncorrected	p-value corrected
HCP-A	323	Level 1	0.99	<0.001	<0.001*	0.64	<0.001	0.021*
	1267	Level 1	0.99	<0.001	<0.001*	0.72	<0.001	<0.001*
		Level 2	0.09	0.63	>0.999	0.03	0.85	>0.999
	1871	Level 1	0.99	<0.001	<0.001*	0.72	<0.001	<0.001*
		Level 2	-0.14	0.45	>0.999	-0.08	0.66	>0.999
HCP-YA	323	Level 1	0.71	<0.001	<0.001*	0.99	<0.001	<0.001*
		Level 2	0.32	0.07	>0.999	-0.00	0.98	>0.999
	1267	Level 1	0.73	<0.001	<0.001*	0.99	<0.001	<0.001*
		Level 2	0.24	0.18	>0.999	-0.26	0.15	>0.999
	1871	Level 1	0.73	<0.001	<0.001*	0.99	<0.001	<0.001*
		Level 2	-0.36	0.04	0.96	0.16	0.39	>0.999
		Level 3	-0.16	0.37	>0.999	-0.48	0.006	0.14

Pearson's correlations between behavioural loadings of the main analyses (granularity level of 1239) in both cohorts with the behavioural loadings of the analyses with other granularity levels in both cohorts. P-values are provided as uncorrected and corrected for multiple comparisons using the Bonferroni method over 24 comparisons. Asterisks indicate significant comparisons.

Supplementary table 6. Comparison between global and modular analyses

Cohort	Analyses and levels	HCP-YA global analysis			HCP-A global analysis		
		r	p-value uncorrected	p-value corrected	r	p-value uncorrected	p-value corrected
HCP-YA modular analysis	CT level 1	0.99	<0.001	<0.001*	0.66	<0.001	<0.001*
	SA level 1	0.99	<0.001	<0.001*	0.74	<0.001	<0.001*
	GMV level 1	0.99	<0.001	<0.001*	0.73	<0.001	<0.001*
HCP-A modular analysis	CT level 1	0.61	<0.001	0.003*	0.98	<0.001	<0.001*
	SA level 1	0.83	<0.001	<0.001*	0.97	<0.001	<0.001*
	SA level 2	0.40	0.02	0.6	0.09	0.62	>0.999
	GMV level 1	0.63	<0.001	0.003*	0.99	<0.001	<0.001*

Pearson’s correlations between behavioural loadings of the global analyses in both cohorts with the behavioural loadings of the modular analyses in both cohorts. P-values are provided as uncorrected and corrected for multiple comparisons using the Bonferroni method over 14 comparisons. Asterisks indicate significant comparisons.

Supplementary figures

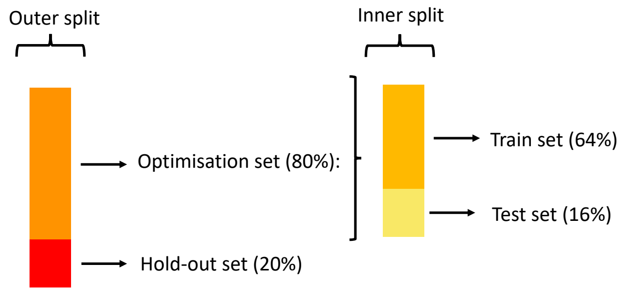

Supplementary figure 1. Machine learning framework. The inner split is used for model selection (train the model finding the regularisation parameters with best generalisability and stability) while the outer split is used for model evaluation (test the generalisability of the selected model).

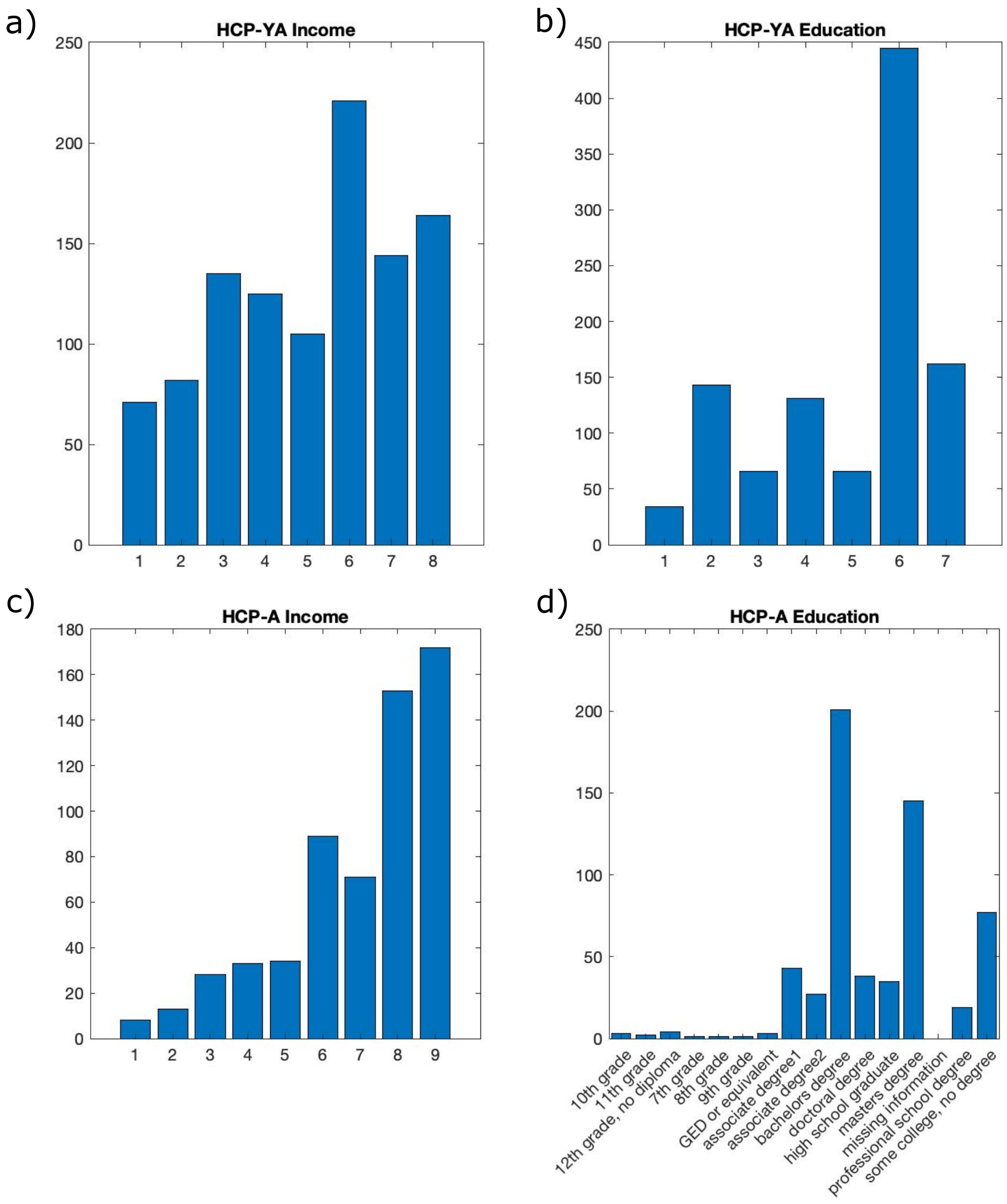

Supplementary figure 2. Demographics of the samples. Income (a,c) and education (b,d) are shown for both cohorts. In both cohorts, values for income correspond to: <$10,000 = 1, 10K-19,999 = 2, 20K-29,999 = 3, 30K-39,999 = 4, 40K-49,999 = 5, 50K-74,999 = 6, 75K-99,999 = 7, >=100,000 = 8. Values in the x-axis for education in HCP-YA (b) correspond to years of education completed, with value 11 corresponding to 11 or less years, and value 17 corresponding to 17 or more years. In the HCP-A cohort (c), the variable household income was converted to categorical ordinal in order to be coherent with the HCP-YA cohort (i.e. values <1000 were replaced by 1, values >1000 & <1999 were replaced by 2, etc). In the bar plot for income in HCP-A (c), value 9 corresponds to missing values.

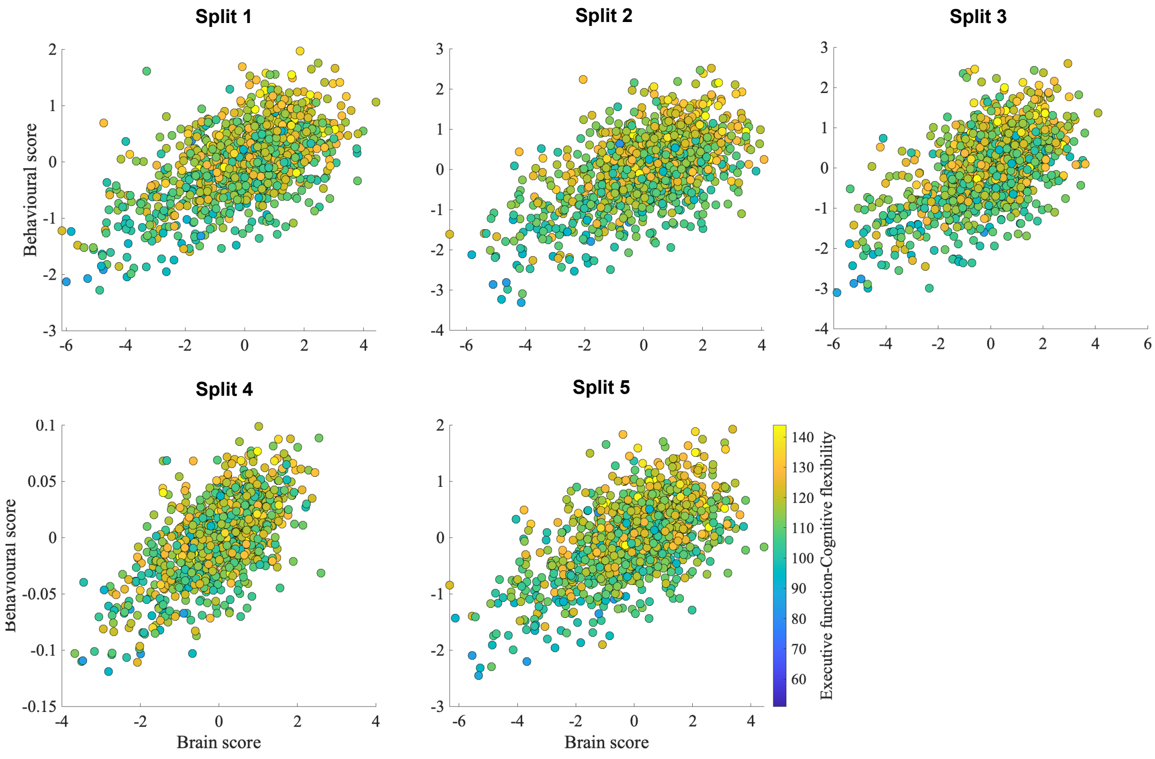

Supplementary figure 3. Latent dimension in all the splits in the HCP-YA cohort. This analysis corresponds to the global analysis in the HCP-YA cohort. Each dot represents one participant.

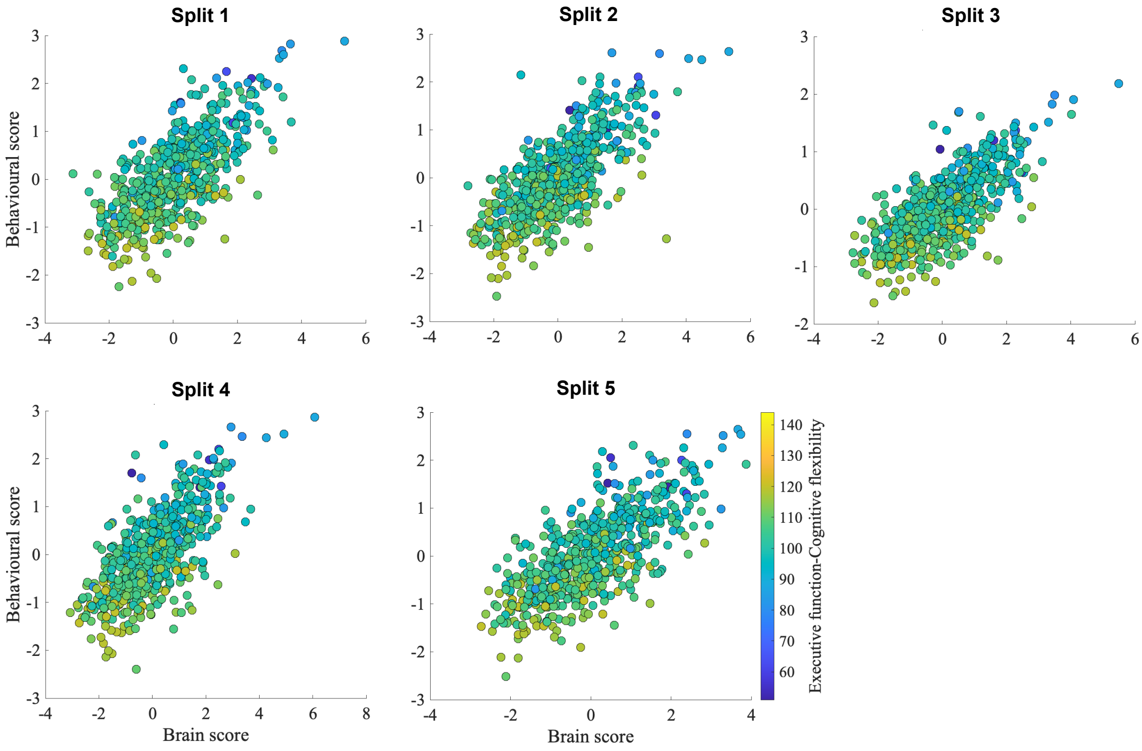

Supplementary figure 4. **Latent dimension in all the splits in the HCP-A cohort.** This analysis corresponds to the global analysis in the HCP-A cohort. Each dot represents one participant.

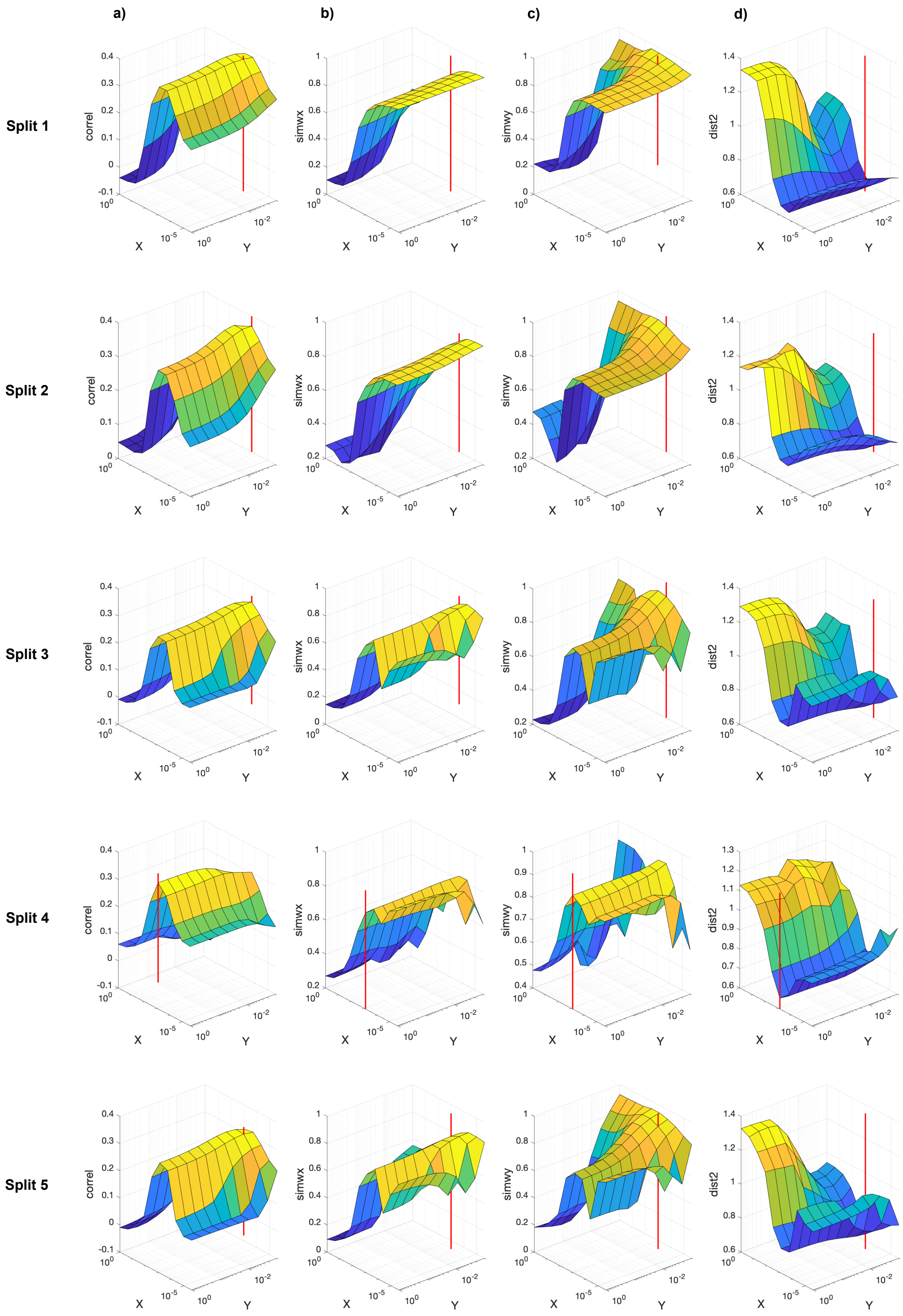

Supplementary figure 5. Model optimisation for the global analysis in all the splits of the HCP-YA cohort. The red line indicates the selected model. The z axis represents the test canonical correlation (column a), the similarity of weights in brain (column b) and behaviour (column c), and the joint generalizability-

stability criteria (column d). The x and y axes represent the hyperparameters tested for brain and behaviour, respectively (in all columns).

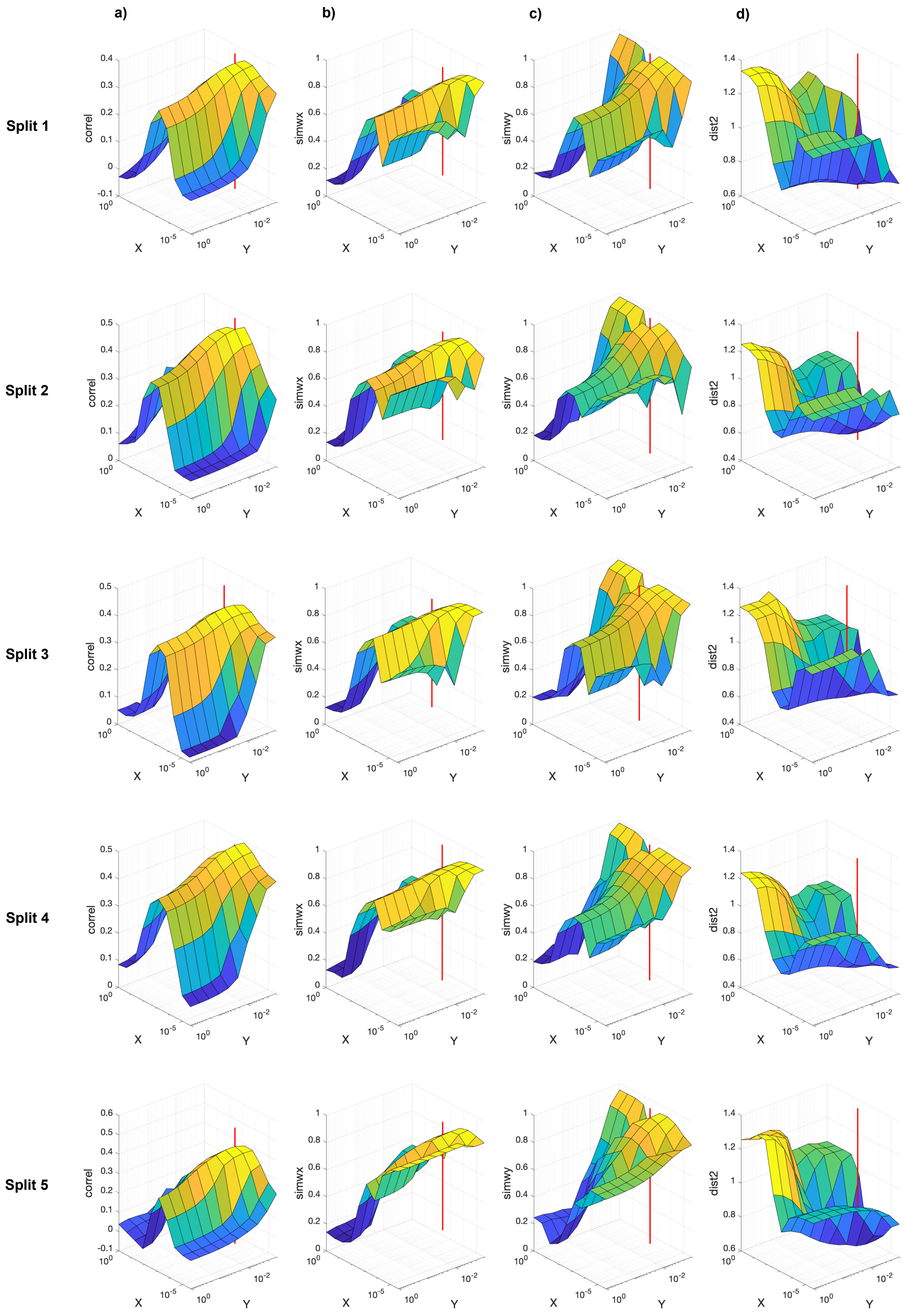

Supplementary figure 6. Model optimisation in the global analysis in all the splits of the HCP-A cohort. The red line indicates the selected model. The z axis represents the test canonical correlation (column a), the similarity of weights in brain (column b) and behaviour (column c), and the joint generalizability-stability

criteria (column d). The x and y axes represent the hyperparameters tested for brain and behaviour, respectively (in all columns).

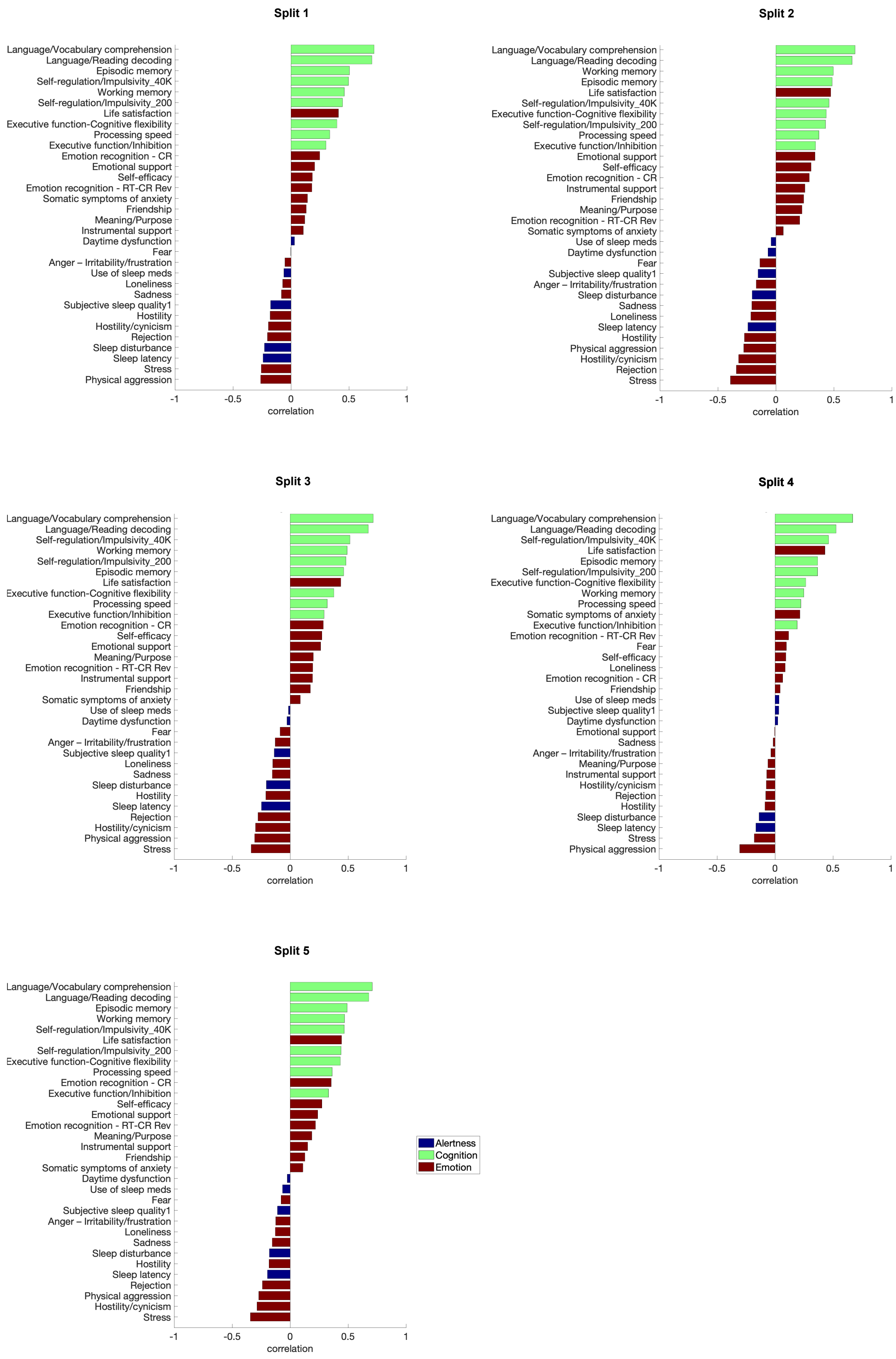

Supplementary figure 7. Behavioural loadings for the global analysis in all the splits of the HCP-YA cohort.

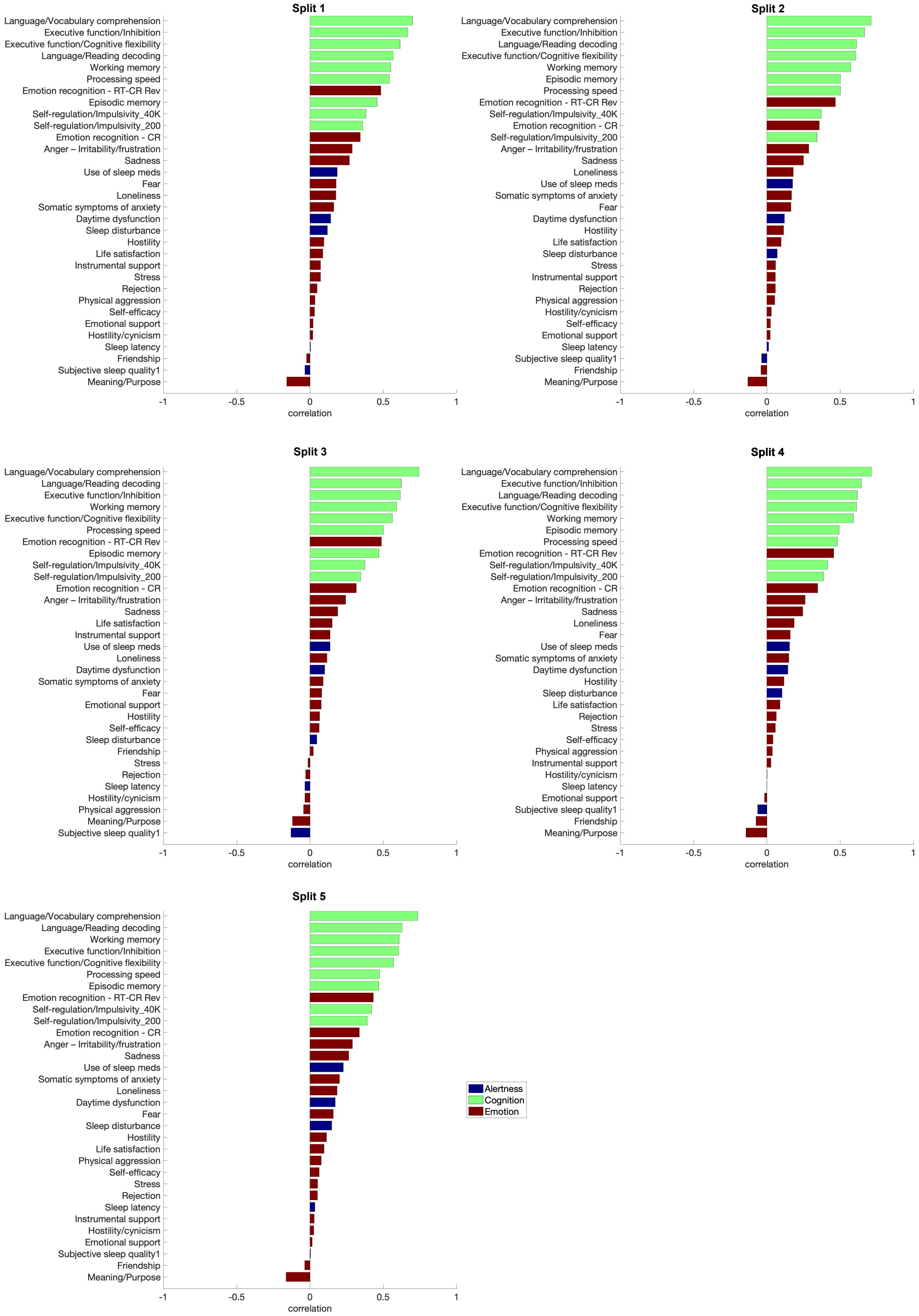

Supplementary figure 8. Behavioural loadings for the global analysis in all the splits of the HCP-A cohort.

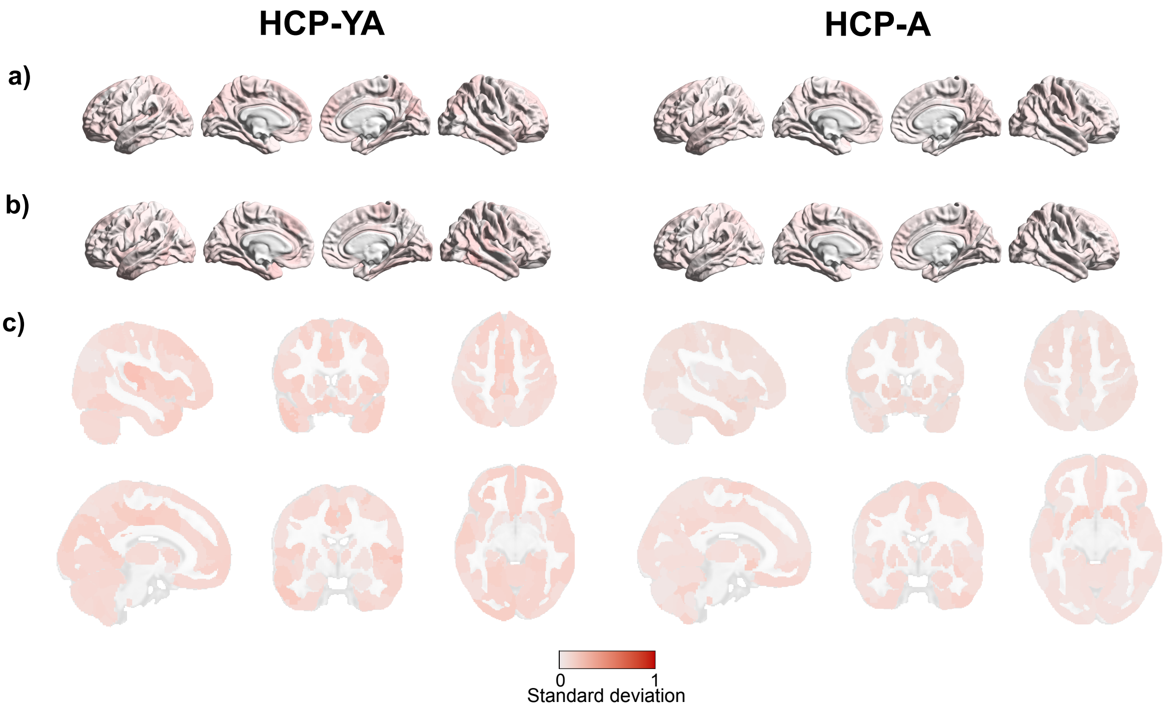

Supplementary figure 9. Standard deviation of brain loadings of global analysis. Standard deviation was computed over the 5 splits. a) Standard deviation for CT. b) Standard deviation for SA. c) Standard deviation for GMV; Top row corresponds to MNI coordinates: -43.6, 16, 52.; Bottom row corresponds to MNI coordinates: -10.3, -3.9, -9.1

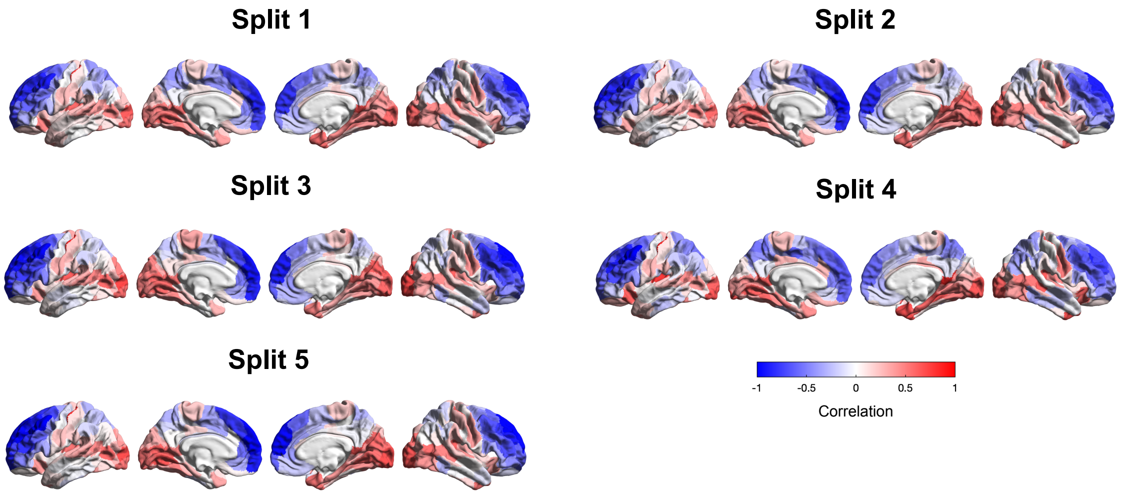

Supplementary figure 10. Cortical thickness loadings for the global analysis in all the splits of the HCP-YA cohort.

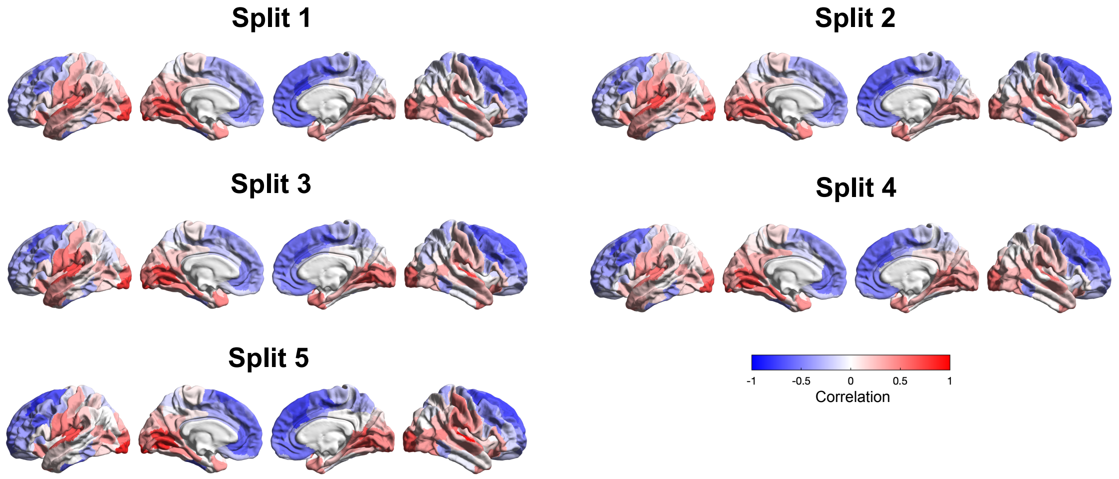

Supplementary figure 11. Cortical thickness loadings for the global analysis in all the splits of the HCP-A cohort.

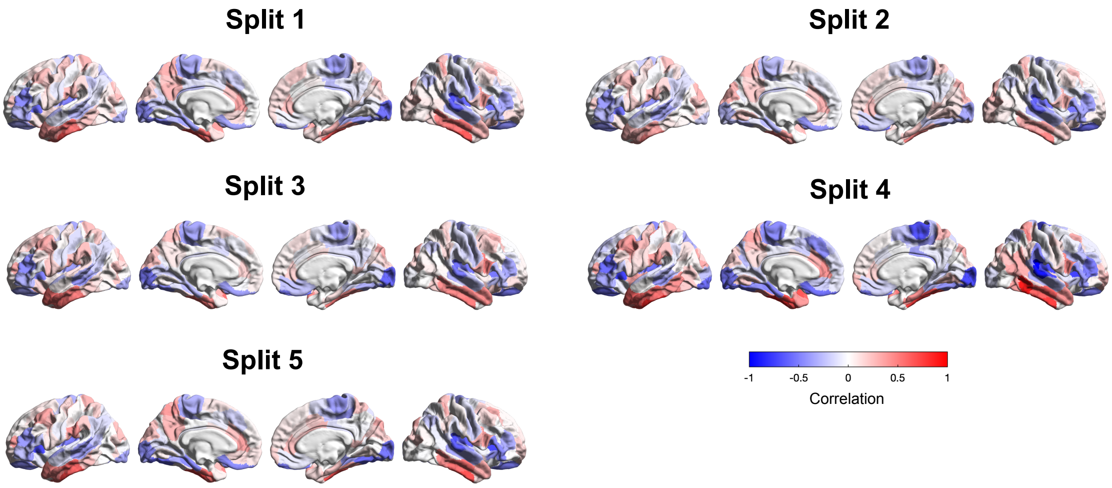

Supplementary figure 12. Surface area loadings for the global analysis in all the splits of the HCP-YA cohort.

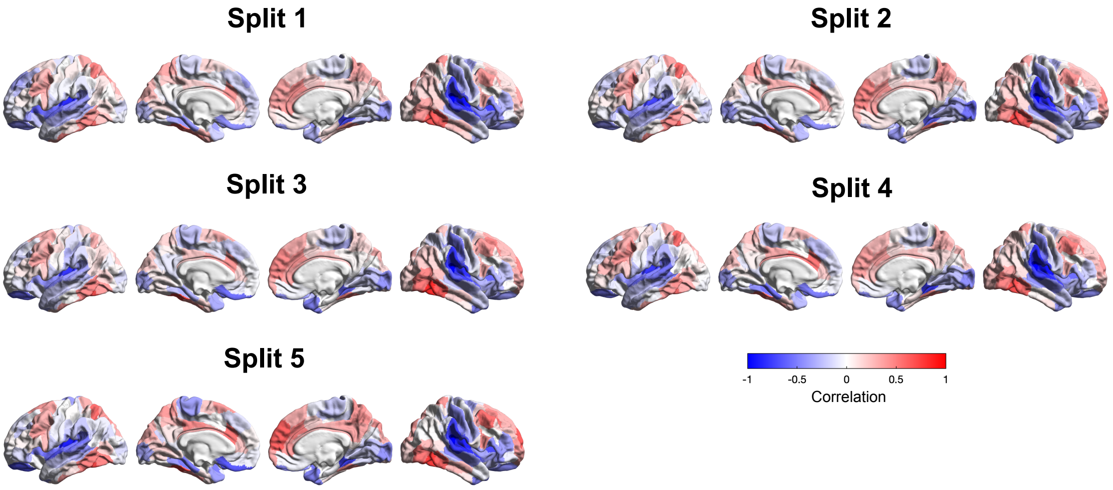

Supplementary figure 13. Surface area loadings for the global analysis in all the splits of the HCP-A cohort.

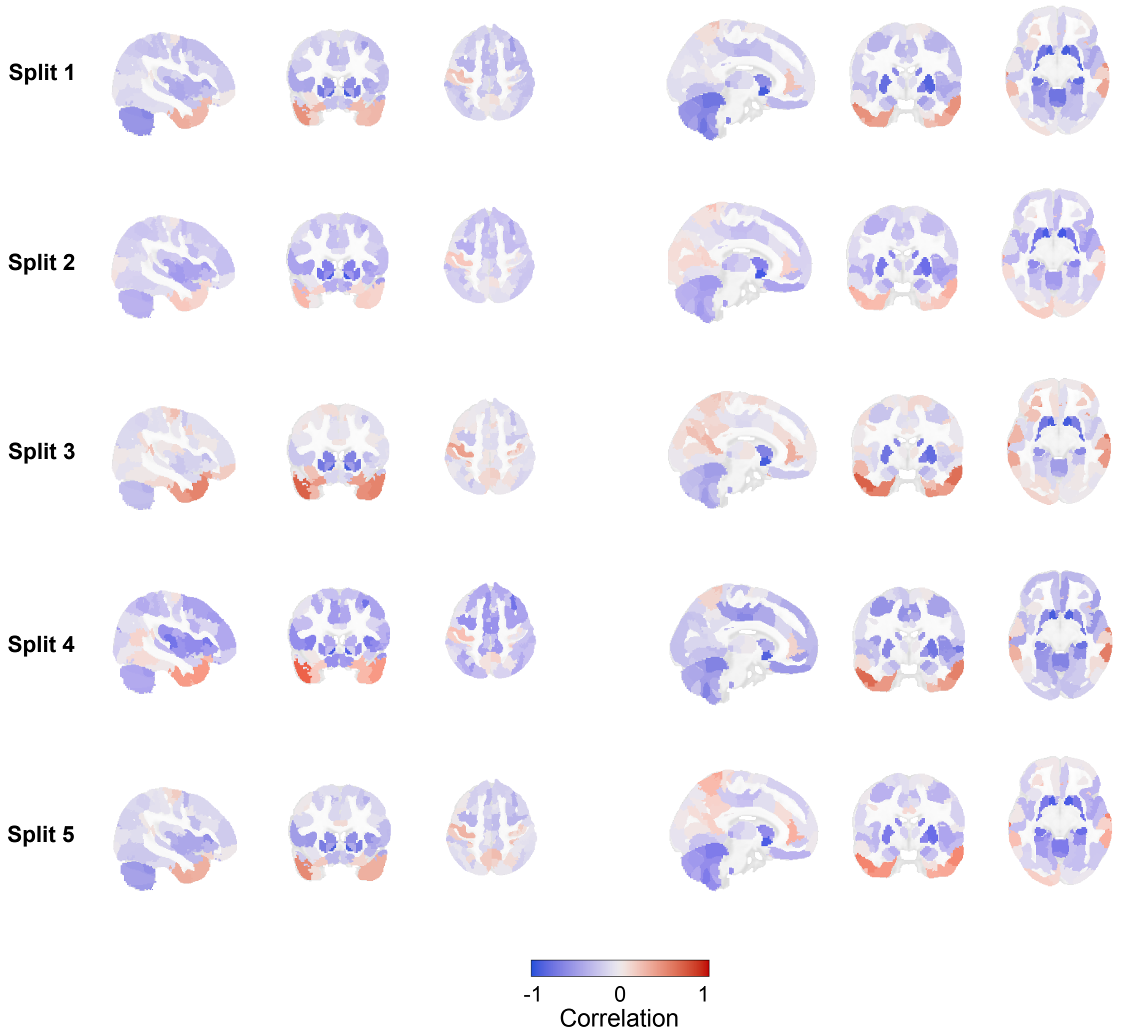

Supplementary figure 14. Grey matter volume loadings for the global analysis in all the splits of the HCP-YA cohort. Left panel corresponds to MNI coordinates: -43.6, 16, 52.9. Right panel corresponds to MNI coordinates: -10.3, -3.9, -9.1.

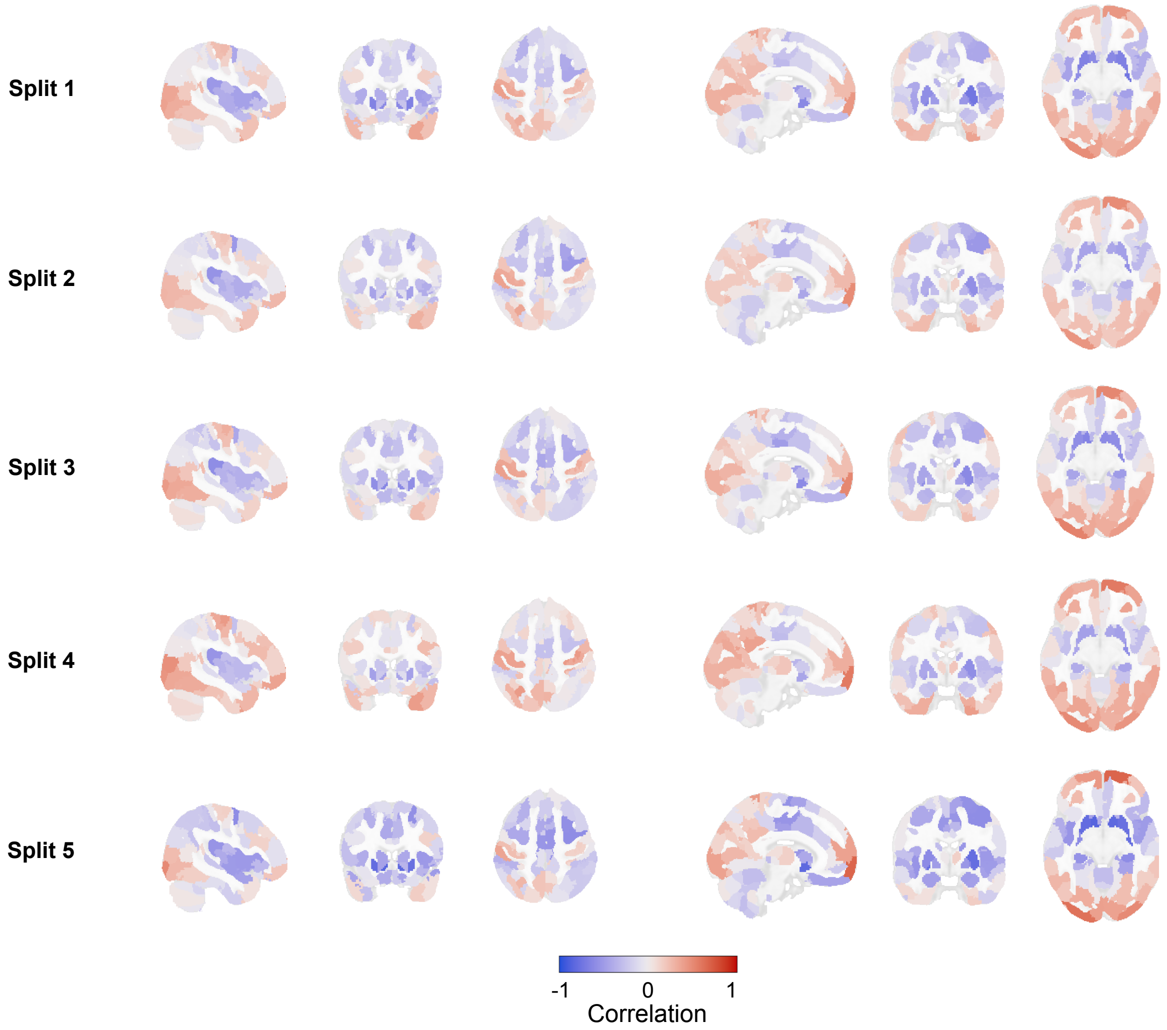

Supplementary figure 15. Grey matter volume loadings for the global analysis in all the splits of the HCP-A cohort. Left panel corresponds to MNI coordinates: -43.6, 16, 52.9. Right panel corresponds to MNI coordinates: -10.3, -3.9, -9.1.

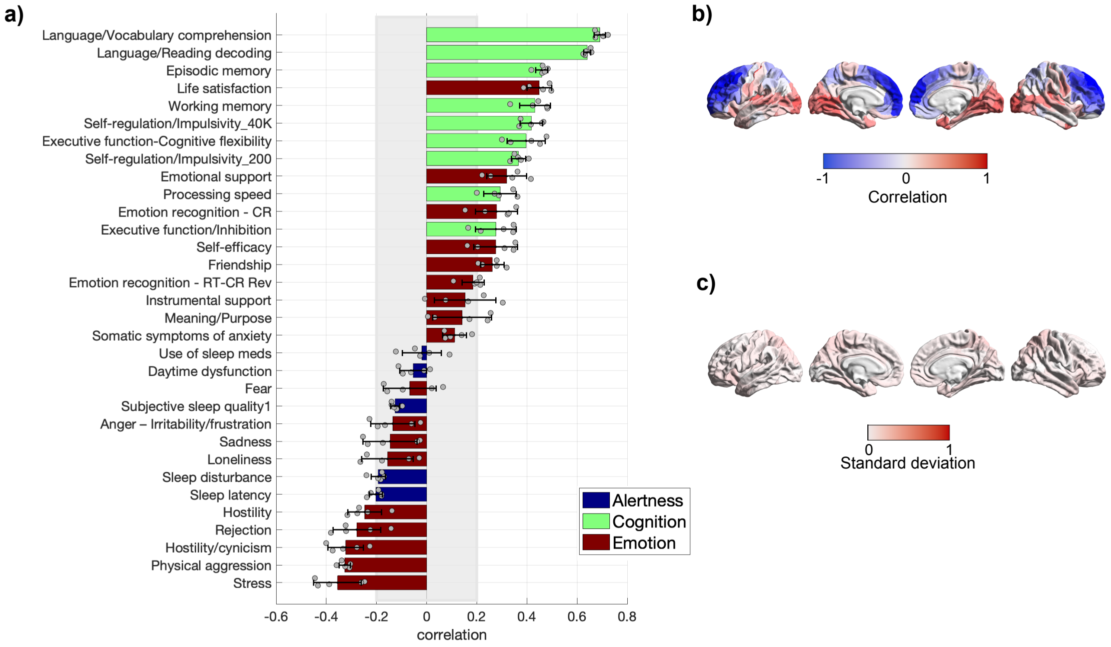

Supplementary figure 16. Loadings of modular analyses for CT in HCP-YA. a) Behavioural loadings, the shadowed zone marks loadings between -0.2 and 0.2. Error bars depict one standard deviation. b) CT loadings. c) Standard deviation of the brain loading across the 5 splits.

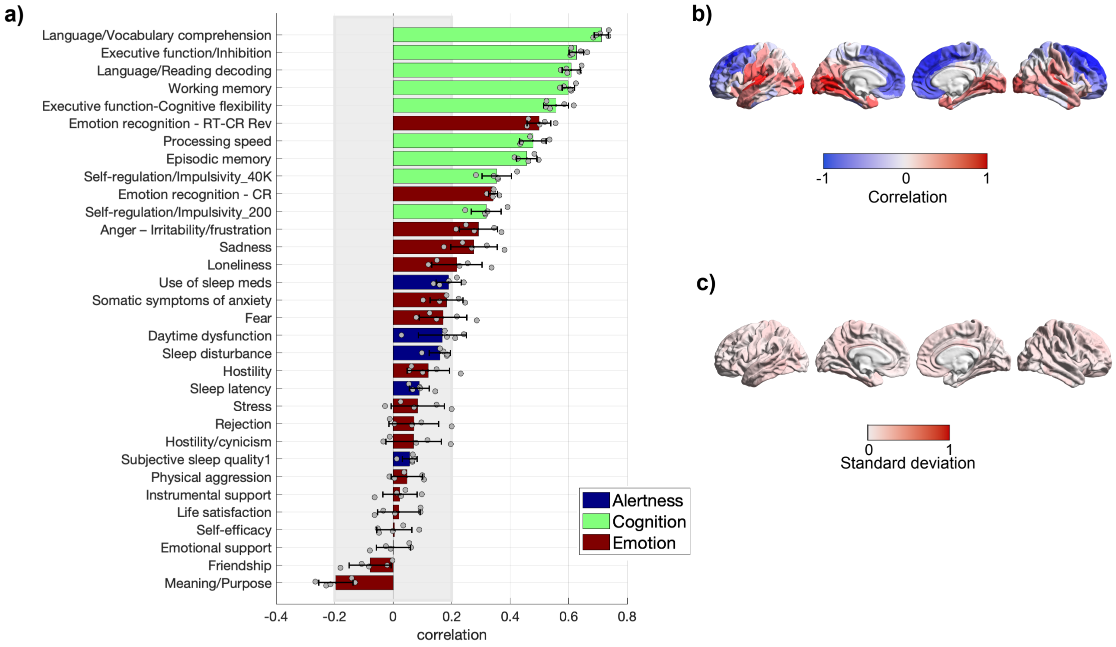

Supplementary figure 17. Loadings of modular analyses for CT in HCP-A. a) Behavioural loadings, the shadowed zone marks loadings between -0.2 and 0.2. Error bars depict one standard deviation. b) CT loadings. c) Standard deviation of the brain loading across the 5 splits.

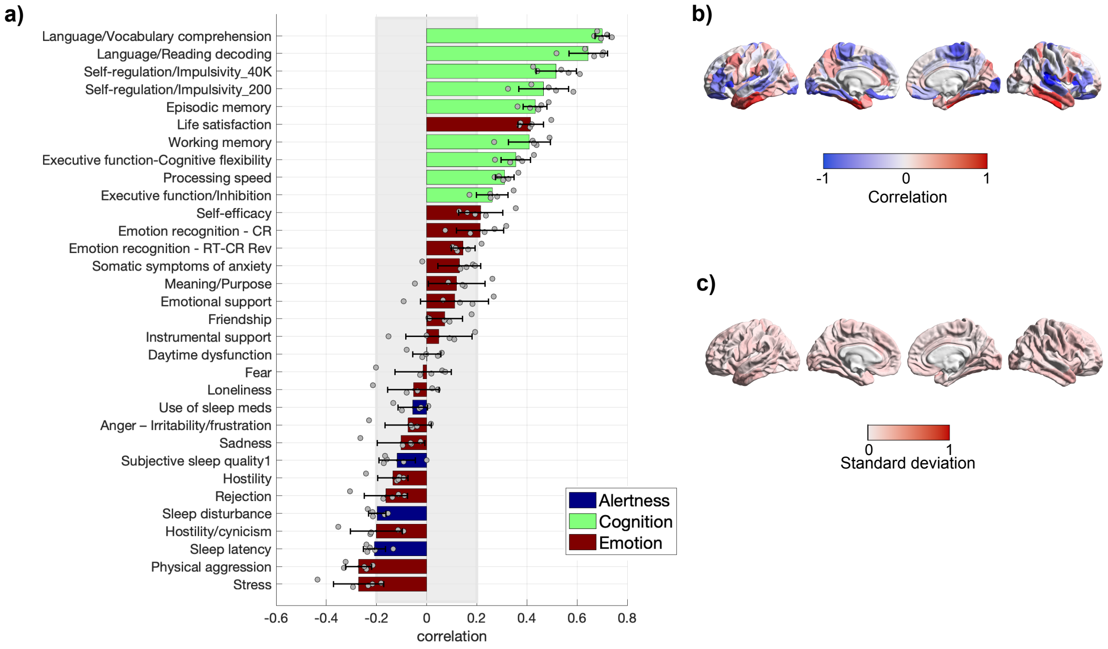

Supplementary figure 18. Loadings of modular analyses for SA in HCP-YA. a) Behavioural loadings, the shadowed zone marks loadings between -0.2 and 0.2. Error bars depict one standard deviation. b) SA loadings. c) Standard deviation of the brain loading across the 5 splits.

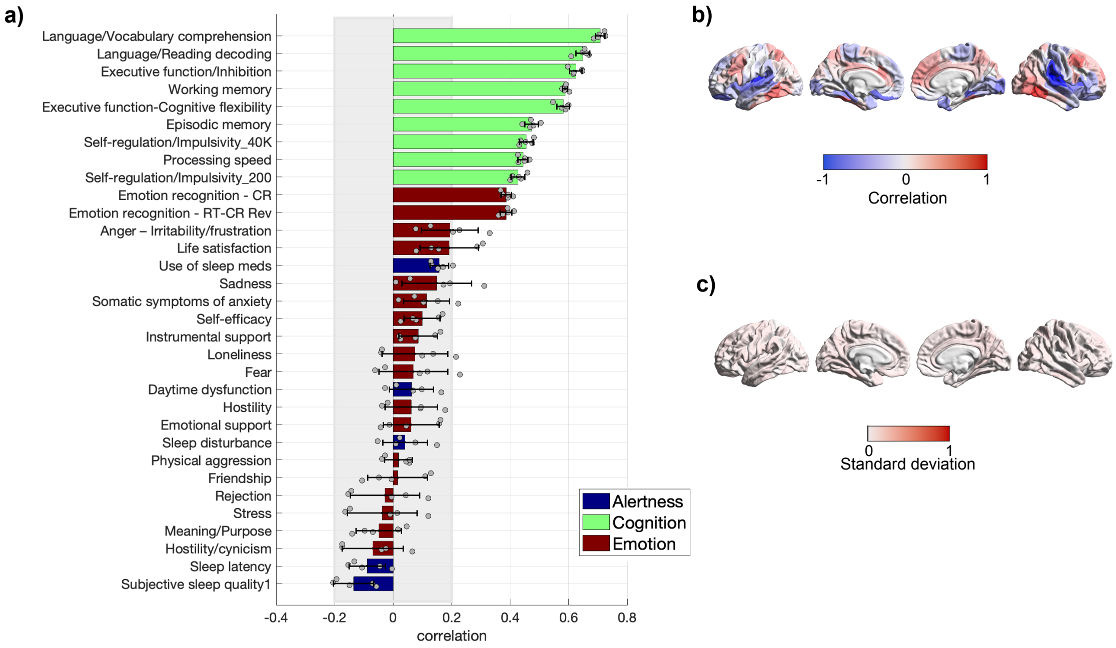

Supplementary figure 19. Loadings of modular analyses for SA in HCP-A. a) Behavioural loadings, the shadowed zone marks loadings between -0.2 and 0.2. Error bars depict one standard deviation. b) SA loadings. c) Standard deviation of the brain loading across the 5 splits.

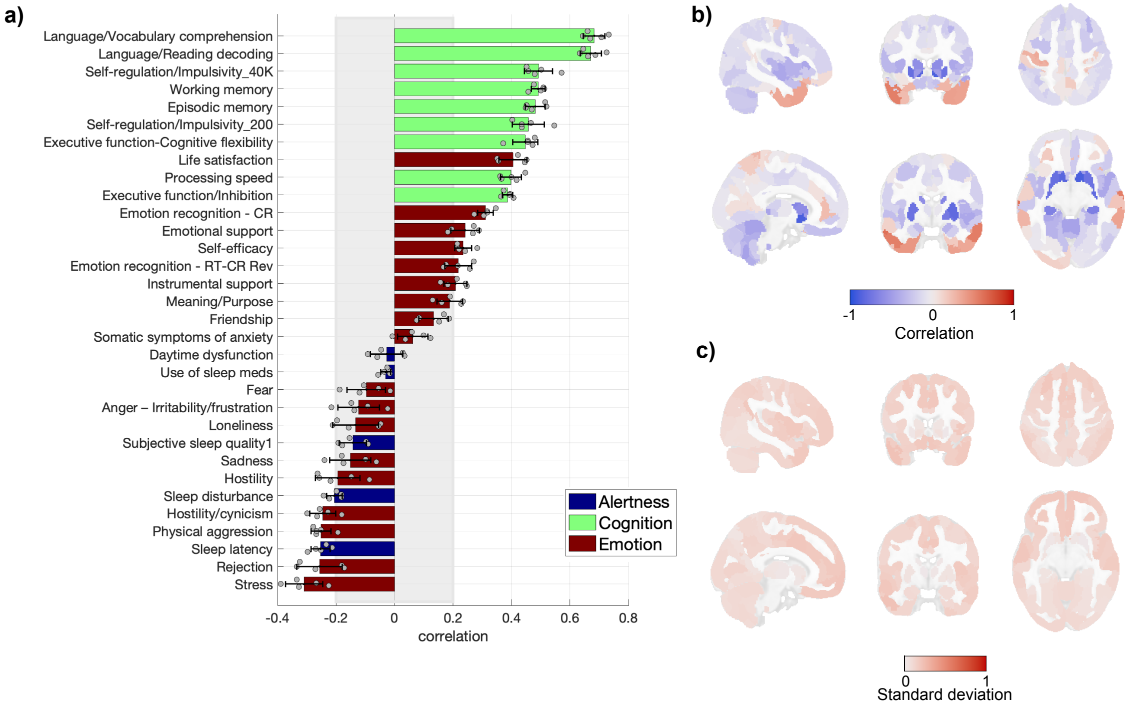

Supplementary figure 20. Loadings of modular analyses for GMV in HCP-YA. a) Behavioural loadings, the shadowed zone marks loadings between -0.2 and 0.2. Error bars depict one standard deviation. b) GMV loadings. Top panel corresponds to MNI coordinates: -43.6, 16, 52.9. Bottom panel corresponds to MNI coordinates: -10.3, -3.9, -9.1. c) Standard deviation of the brain loading across the 5 splits. Top panel corresponds to MNI coordinates: -43.6, 16, 52.9. Bottom panel corresponds to MNI coordinates: -10.3, -3.9, -9.1.

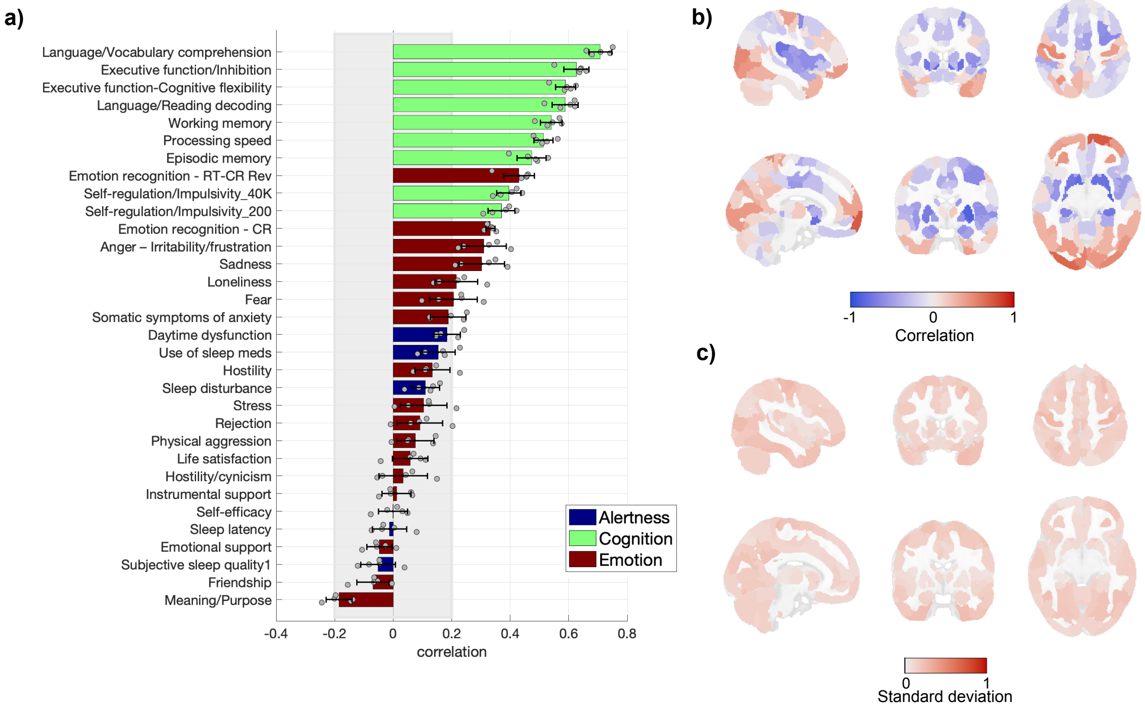

Supplementary figure 21. Loadings of modular analyses for GMV in HCP-A. a) Behavioural loadings, the shadowed zone marks loadings between -0.2 and 0.2. Error bars depict one standard deviation. b) GMV loadings. Top panel corresponds to MNI coordinates: -43.6, 16, 52.9. Bottom panel corresponds to MNI coordinates: -10.3, -3.9, -9.1. c) Standard deviation of the brain loading across the 5 splits. Top panel corresponds to MNI coordinates: -43.6, 16, 52.9. Bottom panel corresponds to MNI coordinates: -10.3, -3.9, -9.1.

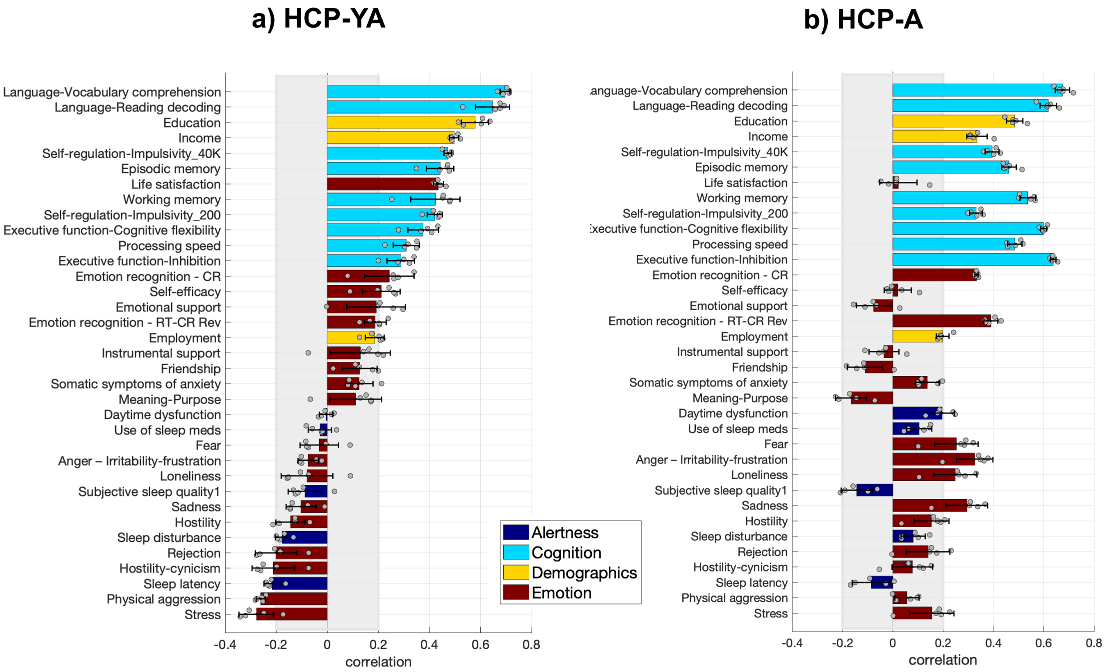

Supplementary figure 22. Behavioural loadings of RCCA linking brain structure to behaviour and socio-economic status a) Behavioural loadings in HCP-YA cohort. b) Behavioural loadings in HCP-A cohort. Shown loadings represent the average over the 5 outer splits. Error bars depict one standard deviation. The shadowed zone marks loadings between -0.2 and 0.2.

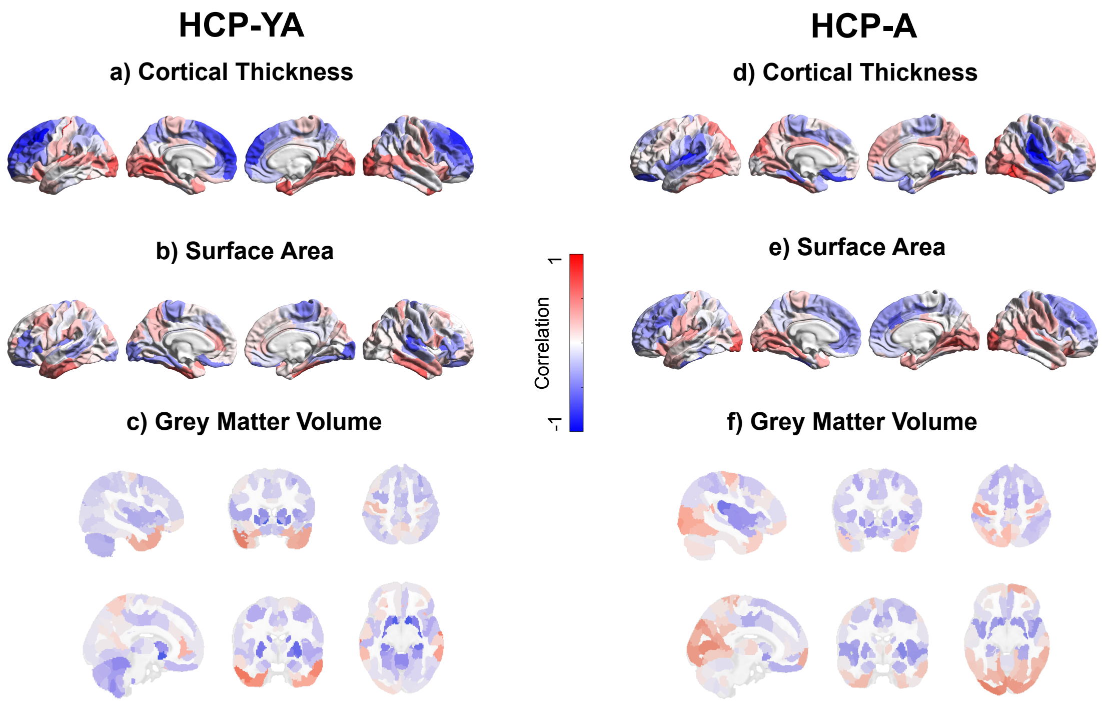

Supplementary figure 23. Brain loadings of RCCA linking brain structure to behaviour and socio-economic status. The left panel shows brain loadings for the HCP-YA cohort, the right panel shows brain loadings for the HCP-A cohort. a,d) Cortical thickness loadings, b,e) Surface area loadings, c,f) Grey matter volume loadings. In panels c and f, top row corresponds to MNI coordinates: -43.6, 16, 52.9; bottom row to MNI coordinates: -10.3, -3.9, -9.1. Shown loadings correspond to the average over the 5 outer splits.

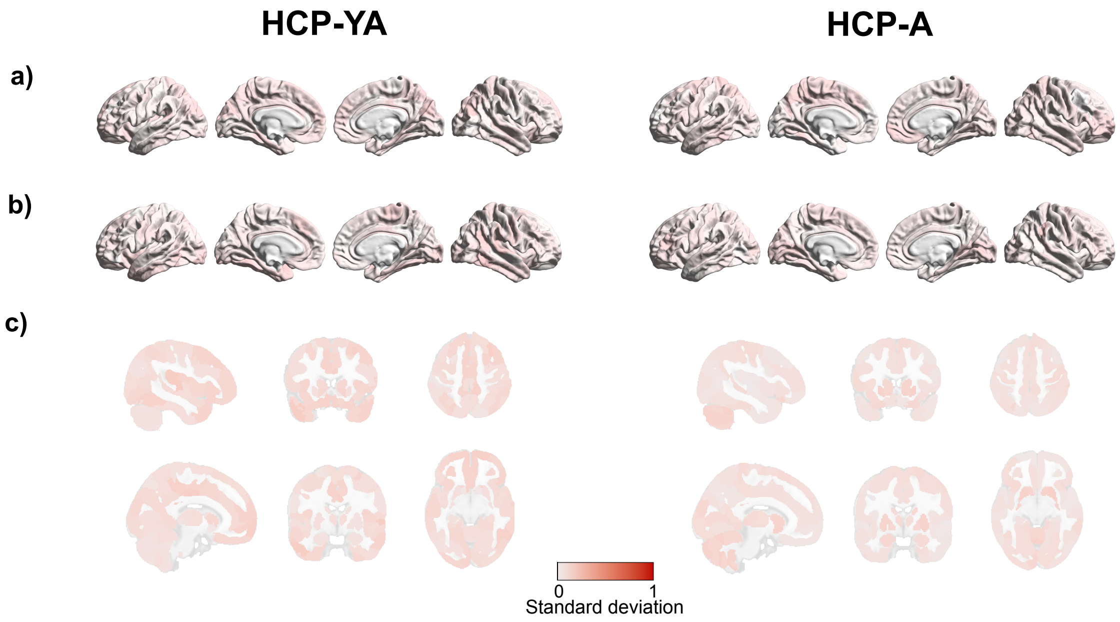

Supplementary figure 24. Standard deviation of brain loadings of RCCA linking brain structure to behaviour and socio-economic status. Standard deviation was computed over the 5 splits. a) Standard deviation for CT. b) Standard deviation for SA. c) Standard deviation for GMV; Top row corresponds to MNI coordinates: -43.6, 16, 52.; Bottom row corresponds to MNI coordinates: -10.3, -3.9, -9.1

Supplementary references

1. Alexander-Bloch, A. F. *et al.* On testing for spatial correspondence between maps of human brain structure and function. *Neuroimage* **178**, 540–551 (2018).

2. Winkler, A. M., Renaud, O., Smith, S. M. & Nichols, T. E. Permutation inference for canonical correlation analysis. *Neuroimage* **220**, 117065 (2020).

3. Schaefer, A. *et al.* Local-Global Parcellation of the Human Cerebral Cortex from Intrinsic Functional Connectivity MRI. *Cereb. Cortex* **28**, 3095–3114 (2018).

4. Tian, Y., Margulies, D. S., Breakspear, M. & Zalesky, A. Topographic organization of the human subcortex unveiled with functional connectivity gradients. *Nat. Neurosci.* **23**, 1421–1432 (2020).

5. Buckner, R. L., Krienen, F. M., Castellanos, A., Diaz, J. C. & Yeo, B. T. T. The organization of the human cerebellum estimated by intrinsic functional connectivity. *J. Neurophysiol.* **106**, 2322–2345 (2011).
