# Supplementary material for: A cross-cohort replicable and heritable latent dimension linking behaviour to multi-featured brain structure
Source: Commun Biol. 2022 Nov 26;5:1297. doi: 10.1038/s42003-022-04244-5 (PMC9701210; doi:10.1038/s42003-022-04244-5)
Supplement: Supplementary file 3 — Description of Additional Supplementary Files [file 42003_2022_4244_MOESM3_ESM.pdf]

Description of Additional Supplementary Files

File name: Supplementary Data 1
Description: Behaviour loadings in HCP-A.

File name: Supplementary Data 2
Description: Brain loadings in HCP-A.

File name: Supplementary Data 3
Description: Behaviour loadings in HCP-YA.

File name: Supplementary Data 4
Description: Brain loadings in HCP-YA.
