# Supplementary material for: A cross-cohort replicable and heritable latent dimension linking behaviour to multi-featured brain structure
Source: Commun Biol. 2022 Nov 26;5:1297. doi: 10.1038/s42003-022-04244-5 (PMC9701210; doi:10.1038/s42003-022-04244-5)
Supplement: Supplementary file 8 — Reporting Summary [file 42003_2022_4244_MOESM8_ESM.pdf]

nature portfolio

Corresponding author(s): Eliana Nicolaisen-Sobesky, Dr. Sarah Genon

Last updated by author(s): November 1st, 2022

Reporting Summary

Nature Portfolio wishes to improve the reproducibility of the work that we publish. This form provides structure for consistency and transparency in reporting. For further information on Nature Portfolio policies, see our Editorial Policies and the Editorial Policy Checklist.

Statistics

For all statistical analyses, confirm that the following items are present in the figure legend, table legend, main text, or Methods section.

n/a	Confirmed	
☐	☒	The exact sample size (n) for each experimental group/condition, given as a discrete number and unit of measurement
☐	☒	A statement on whether measurements were taken from distinct samples or whether the same sample was measured repeatedly
☐	☒	The statistical test(s) used AND whether they are one- or two-sided<br>*Only common tests should be described solely by name; describe more complex techniques in the Methods section.*
☐	☒	A description of all covariates tested
☐	☒	A description of any assumptions or corrections, such as tests of normality and adjustment for multiple comparisons
☐	☒	A full description of the statistical parameters including central tendency (e.g. means) or other basic estimates (e.g. regression coefficient) AND variation (e.g. standard deviation) or associated estimates of uncertainty (e.g. confidence intervals)
☒	☐	For null hypothesis testing, the test statistic (e.g. F, t, r) with confidence intervals, effect sizes, degrees of freedom and P value noted<br>*Give P values as exact values whenever suitable.*
☒	☐	For Bayesian analysis, information on the choice of priors and Markov chain Monte Carlo settings
☒	☐	For hierarchical and complex designs, identification of the appropriate level for tests and full reporting of outcomes
☐	☒	Estimates of effect sizes (e.g. Cohen's d, Pearson's r), indicating how they were calculated

Our web collection on statistics for biologists contains articles on many of the points above.

Software and code

Policy information about availability of computer code

Data collection	All data analyzed in this manuscript were obtained from the open-access HCP young adult and HCP aging samples
Data analysis	MATLAB R2020b and python3 were used for data curation; the RCCA analyses and the machine learning framework were implemented in MATLAB R2020b, Heritability and genetic correlations analyses were implemented in SOLAR Eclipse version 8.5.1; Computational Anatomy Toolbox version 12.5 was used to estimate grey matter volume. Cortical thickness and surface area were obtained by HCP using FreeSurfer version 5.3.0-HCP and FreeSurfer version 6.0 for HCP-young adult and HCP-aging, respectively.

For manuscripts utilizing custom algorithms or software that are central to the research but not yet described in published literature, software must be made available to editors and reviewers. We strongly encourage code deposition in a community repository (e.g. GitHub). See the Nature Portfolio guidelines for submitting code & software for further information.

Data

Policy information about availability of data

All manuscripts must include a data availability statement. This statement should provide the following information, where applicable:

- Accession codes, unique identifiers, or web links for publicly available datasets
- A description of any restrictions on data availability
- For clinical datasets or third party data, please ensure that the statement adheres to our policy

Access to data of the HCP can be requested on ConnectomeDB (https://db.humanconnectome.org/app/template/Login.vm).

Human research participants

Policy information about studies involving human research participants and Sex and Gender in Research.

Reporting on sex and gender	Gender data was acquired as self-report. Age and gender were regressed out from the data.
Population characteristics	The Human Connectome Project - Young Adult sample has an age range of 22-37 years old, with mean age of 28.78 and standard deviation of 3.67 years. The Human Connectome Project - Aging sample has an age range of 36-100 years old, with mean age of 58.5 and standard deviation of 14.9 years.
Recruitment	Recruitment has been done by the Human Connectome Project (https://www.humanconnectome.org).
Ethics oversight	Heinrich Heine University Düsseldorf ethics committee

Note that full information on the approval of the study protocol must also be provided in the manuscript.

Field-specific reporting

Please select the one below that is the best fit for your research. If you are not sure, read the appropriate sections before making your selection.

☒ Life sciences ☐ Behavioural & social sciences ☐ Ecological, evolutionary & environmental sciences

For a reference copy of the document with all sections, see nature.com/documents/nr-reporting-summary-flat.pdf

Life sciences study design

All studies must disclose on these points even when the disclosure is negative.

Sample size	We used two publicly available and large-scale datasets of the Human Connectome Project (HCP): the HCP Young Adult (HCP-YA, S1200 release) and the HCP in Aging (HCP-A, 2.0 release).<br><br>The HCP-YA cohort comprises neuroimaging and behavioural data of 1206 volunteers between 22-37 years old. Subjects are healthy individuals born in Missouri to families that include twins. The sample consists of 457 families, including 292 monozygotic twins, 323 dizygotic twins and 586 not-twins. We excluded 93 subjects for not having available structural scans, 2 for errors during CAT processing and 66 for not having complete data, leading to a final sample of 1047 subjects (560 females, mean age=28.78 years, SD age=3.67 years, age range=22-37 years). The final sample included 94 subjects with ethnicity Hispanic/Latino, 940 with ethnicity Not Hispanic/Latino, and 13 with unknown or not reported ethnicity.<br><br>The HCP-A cohort includes neuroimaging and behavioural data of 725 healthy adults between 36 to 100 years old. We excluded 1 subject for technical problems, 5 subjects for errors in the CAT processing (estimated untypical tissue peaks) and 118 for not having complete behavioural data. This leads to a final sample of 601 unrelated subjects (353 females, mean age=58.5 years, SD age=14.9 years, age range=36-100 years). The final sample included 65 subjects with ethnicity Hispanic/Latino, 535 with ethnicity Not Hispanic/Latino, and 1 with unknown or not reported ethnicity. Subjects of this sample included in this study were unrelated (did not pertain to the same families).
Data exclusions	In the HCP-YA we excluded 93 subjects for not having available structural scans, 2 for errors during CAT processing and 66 for not having complete data. In the HCP-A we excluded 1 subject for technical problems, 5 subjects for errors in the CAT processing (estimated untypical tissue peaks) and 118 for not having complete behavioural data
Replication	The replicability of the latent dimensions was tested by comparing the mean brain and behavioural loadings across cohorts. Loadings of each latent dimension in each cohort were averaged over the 5 outer splits. Behavioural loadings were compared across cohorts with Pearson correlation. The CT and SA loadings were compared across cohorts using spin test, to account for their spatial dependencies
Randomization	Our study does not include experimental groups, so no randomization was done.
Blinding	Our study does not include experimental groups, so no blinding was done.

Reporting for specific materials, systems and methods

We require information from authors about some types of materials, experimental systems and methods used in many studies. Here, indicate whether each material, system or method listed is relevant to your study. If you are not sure if a list item applies to your research, read the appropriate section before selecting a response.

Materials & experimental systems

n/a	Involved in the study
☒	☐ Antibodies
☒	☐ Eukaryotic cell lines
☒	☐ Palaeontology and archaeology
☒	☐ Animals and other organisms
☒	☐ Clinical data
☒	☐ Dual use research of concern

Methods

n/a	Involved in the study
☒	☐ ChIP-seq
☒	☐ Flow cytometry
☐	☒ MRI-based neuroimaging

Magnetic resonance imaging

Experimental design

Design type: Structural imaging (T1)

Design specifications: not relevant

Behavioral performance measures: We selected those behavioural variables focused on emotion and cognition that were present in both cohorts without missing values. The selected behavioural variables spanned sleep, episodic memory, executive functions, language, processing speed, self-regulation/impulsivity, working memory, emotion recognition, negative affect, psychological well-being, social relationships, and stress and self-efficacy (see supplementary file 1 for specific behavioural variables included). 32 behavioural variables were used.

Acquisition

Imaging type(s): structural

Field strength: 3T

Sequence & imaging parameters: Neuroimaging data in the HCP-YA cohort were obtained using a customised 3T Magnetic Resonance Siemens Skyra “Connectom” scanner with a standard 32-channel Siemens receive head coil in a single site at Washington University in St. Louis, United States of America. T1-weighted images were obtained using a 3D MPRAGE sequence (TR = 2400 ms; TE = 2.14 ms; TI = 1000 ms; voxel size = 0.7 mm isotropic). In the HCP-A cohort, neuroimaging data were acquired on standard Siemens 3T Prisma scanners with Siemens 32-channel Prisma head coils at four sites in the United States of America: Washington University in St. Louis, University of California-Los Angeles, University of Minnesota and Massachusetts General Hospital [48]. Matched neuroimaging protocols were used across sites. T1-weighted images were obtained using multi-echo MPRAGE sequences (TR/TI = 2500/1000; TE = 1.8/3.6/5.4/7.2 ms; voxel size = 0.8 mm isotropic).

Area of acquisition: whole-brain

Diffusion MRI: ☐ Used ☒ Not used

Preprocessing

Preprocessing software: Computational Anatomy Toolbox version 12.5 was used to estimate grey matter volume. Cortical thickness and surface area were obtained by HCP using FreeSurfer version 5.3.0-HCP and FreeSurfer version 6.0 for HCP-young adult and HCP-aging, respectively.

Normalization: The T1-w anatomical images of both cohorts were processed with the Computational Anatomy Toolbox version 12.5. After normalization and segmentation, the grey matter segments were modulated for non-linear transformations and smoothed. Grey matter was parcellated using a combination of the Schaefer atlas for 200 cortical regions, the Melbourne subcortex atlas for 32 subcortical regions and the Buckner/Yeo atlas for 7 cerebellar regions. CT and SA were obtained from the HCP, estimated with FreeSurfer version 5.3.0-HCP in HCP-YA and with version 6.0 in HCP-A. CT and SA were parcellated using the Schaefer atlas for 200 regions .

Normalization template: MNI

Noise and artifact removal: not relevant

Volume censoring: not relevant

Statistical modeling & inference

Model type and settings: multivariate: regularized canonical correlation analysis

Effect(s) tested: canonical correlation between behaviour and brain structure.
replication of the latent dimension across cohorts, by Pearson's correlation and spin test of behavioural, CT and SA loadings.

Comparison of the CT and SA loadings with the principal gradient of functional connectivity usin spin test.
Heritability and genetic correlation of brain and behavioural scores

Specify type of analysis: ☒ Whole brain ☐ ROI-based ☐ Both

Statistic type for inference (See Eklund et al. 2016): multivariate, including 32 behavioural variables and 639 roi-wise brain structural measures

Correction: Bonferroni

Models & analysis

n/a	Involved in the study
☒	☐ Functional and/or effective connectivity
☒	☐ Graph analysis
☐	☒ Multivariate modeling or predictive analysis

Multivariate modeling and predictive analysis

Grey matter was parcellated using a combination of the Schaefer atlas for 200 cortical regions, the Melbourne subcortex atlas for 32 subcortical regions and the Buckner/Yeo atlas for 7 cerebellar regions. CT and SA were parcellated using the Schaefer atlas for 200 regions.
Model used was Regularized canonical correlation analysis.
We used a recently proposed machine learning framework that uses multiple holdouts of the data. In this framework, two consecutive splits of the data are used for model selection and statistical evaluation. The first split (i.e., outer split) creates an optimisation set (80%) and a hold-out set (20%). The second split (i.e., inner split) divides the optimisation set into training set (80% of the optimisation set) and testing set (20% of the optimisation set). Several RCCA models, each with a different combination of regularisation parameters, are fitted on the training sets. Then the testing sets are projected onto the obtained weights, yielding test canonical correlations. The combination of parameters yielding the highest test canonical correlation is then selected as best regularisation parameters and used to fit the whole optimisation set. Finally, the hold-out set is projected onto the weights obtained in the optimisation set in order to test for the generalisability of the model. Hence, several splits of the data are used to create multiple hold-out datasets. This means that several hold-out datasets are used, which assesses the robustness of the model. The data partitions respected the family structure of the HCP-YA cohort. We used 5 outer splits and 5 inner splits.
